# Response to Pneumococcal Polysaccharide Vaccination in Newly Diagnosed HIV-Positive Individuals

**DOI:** 10.4172/2155-6113.1000419

**Published:** 2015-01-23

**Authors:** David J Leggat, Anita S Iyer, Jennifer A Ohtola, Sneha Kommoori, Joan M Duggan, Claudiu A Georgescu, Sadik A Khuder, Noor M Khaskhely, MA Julie Westerink

**Affiliations:** 1 Department of Medicine, University of Toledo, USA; 2 Department of Medical Microbiology and Immunology, University of Toledo, USA; 3 Department of Internal Medicine, University of Toledo, USA; 4 Department of Pathology, University of Toledo, USA; 5 Department of Physiology, University of Toledo, USA; 6 Department of Pharmacology, University of Toledo, USA; 7 Department of Metabolism & Cardiovascular Science, University of Toledo, USA; 8 Department of Public Health, University of Toledo, USA

**Keywords:** B cell, Pneumococcus, Streptococcus pneumonia, HIV, Vaccine, Polysaccharide, Antigen-specific

## Abstract

**Background:**

Newly diagnosed HIV-positive individuals are 35 to 100-fold more susceptible to *Streptococcus pneumoniae* infection compared to non-infected individuals. Therefore, the 23-valent pneumococcal polysaccharide vaccine (PPV23) has previously been recommended, though efficacy and effectiveness of vaccination remains controversial. Early severe B cell dysfunction is a central feature of HIV infection. The specific nature of the immune cells involved in the production of protective antigen-specific antibodies in HIV-positive individuals remains to be elucidated.

**Objectives:**

Evaluate the antibody and antigen-specific B cell response to the 23-valent pneumococcal polysaccharide vaccine in newly diagnosed HIV-positive patients. Moreover, determine if newly diagnosed patients with CD4<200 cells/μl benefit from 6–12 months of HAART, allowing partial viral suppression and immune reconstitution, prior to immunization.

**Methods:**

Newly diagnosed HIV-positive patients with CD4>200 cells/μl and CD4<200 cells/μl were immunized with PPV23. Patients with CD4<200 cells/μl received either immediate or delayed immunization following 6–12 months of HAART. Antibody responses, opsonophagocytic activity and phenotypic analysis of pneumococcal polysaccharide-specific B cells were studied.

**Results:**

Newly diagnosed HIV-positive patients demonstrated CD4-dependent increases in antibody and opsonophagocytic titers thought to be commensurate with protection. Functional opsonophagocytic titers of patients with CD4<200 cells/μl immunized immediately compared to patients with CD4<200 cells/μl receiving HAART for 6–12 months were not significantly different. Pneumococcal polysaccharide-specific B cells were distributed evenly between IgM memory and switched memory B cells for all groups, but IgM memory B cells were significantly lower than in HIV-negative individuals.

**Conclusions:**

Despite CD4-dependent pneumococcal polysaccharide-specific deficiencies in newly diagnosed HIV-positive patients, vaccination was beneficial based on opsonophagocytic titers for all newly diagnosed HIV-positive groups. In HIV-positive patients with CD4<200 cells/μl, 6–12 months of HAART did not improve opsonophagocytic titers or antibody concentrations. Based on these findings, immunization with the 23-valent pneumococcal polysaccharide vaccine should not be delayed in newly diagnosed HIV-positive patients with CD4<200 cells/μl.

## Introduction

Newly diagnosed HIV-positive individuals are 35 to 100-fold more susceptible to invasive *Streptococcus pneumoniae* infection compared to HIV-negative individuals [1,2]. Pneumococcus is the most common bacterial respiratory pathogen in HIV-positive individuals and a major cause of morbidity and mortality requiring hospitalized care [3,4]. Incidence of invasive pneumococcal disease in individuals not receiving antiretroviral therapy has been reported to be 281 per 100,000 individuals [5]. The 23-valent pneumococcal polysaccharide vaccine (PPV23) has previously been recommended for all HIV-positive adults by the Advisory Committee for Immunization Practices (ACIP), though efficacy and effectiveness of vaccination remains controversial [3,6,7].

Vaccine response to PPV23 is measured by testing antibody levels via enzyme-linked immunosorbant assay (ELISA) and opsonophagocytic assay which represent immunological correlates of protection. It should be noted that opsonophagocytic titers are thought to be a more accurate surrogate of protection while antibody titers correspond poorly to protection. Although protective levels for these correlates are not well defined in adults, they are suboptimal compared to HIV-negative individuals and correlate with patient CD4 counts [8,9]. To provide better therapeutic treatment, a better understanding of intrinsic B cell defects resulting from HIV infection that lead to increased pneumococcal disease incidence is critical for the development of a more efficacious vaccine.

HIV-positive patients are often unaware of their initial contraction of the HIV virus. Therefore, it is common for HIV-positive patients to be newly diagnosed at various stages of infection, and CD4 counts are used as a surrogate marker for disease progression and immune suppression. In addition, early severe B cell dysfunction is a central feature of HIV infection [6,10,11]. Overall, the total number of memory B cells is reduced in HIV-positive individuals [11–13]. In addition, HIV infection causes B cell polyclonal activation, hypergammaglobulinemia, and high spontaneous antibody production *in vitro* during early stages of disease before quantitative and qualitative defects in CD4+T cells occur, suggesting intrinsic B cell defects [14–18]. This results in the production of excessive but non-functional antibodies [19]. Conversely, functional anti-pneumococcal IgM and IgG antibodies critical for bacterial clearance are severely reduced in HIV-positive individuals immunized with PPV23 compared to HIV-negative individuals [20–22]. This suggests that HIV-positive individuals lack important pneumococcal polysaccharide (PPS) responding B cell subsets necessary to provide sufficient protection. The specific nature of the immune cells involved in the production of protective antigen-specific antibodies in HIV-positive individuals remains to be elucidated.

There were three goals in this study. First, to elucidate the immunogenic response to PPV23 in newly diagnosed HIV-positive individuals. Second, to evaluate whether it is potentially beneficial to provide 6–12 months of HAART (highly active anti-retroviral therapy) to suppress viral load and potentially improve immune function before PPV23 vaccination in newly diagnosed HIV-positive individuals with CD4<200. Third, to elucidate the phenotypic distribution of PPS-selected B cells in newly diagnosed HIV-positive individuals, dependent on CD4 count, compared to HIV-negative individuals. Data supporting vaccination recommendations for HIV-positive individuals with CD4<200 remain to be elucidated. It is not known if newly-diagnosed HIV-positive individuals with CD4<200 benefit from delayed immunization following 6–12 months HAART allowing viral suppression and partial immune reconstitution.

## Methods

### Study population and design

Forty-three pneumococcal polysaccharide vaccine naïve newly diagnosed HIV-positive volunteers participated in the University of Toledo IRB committee approved open observational study (IRB # 106410 and 107017). Volunteers were recruited between 2011 and 2014 at the University of Toledo Medical Center. All volunteers were surveyed for exclusion criteria including: pregnancy, cancer, autoimmune disease, bleeding disorders, recently receiving blood products, previous pneumococcal vaccination, organ transplantation, splenectomy, end stage renal or liver disease, and immunosuppressive medications. Informed consent was obtained from all participants. Volunteer baseline information also included age, gender, ethnicity, treatment with HAART, patient history, hepatitis status, list of medications, physical exams, and blood tests (complete blood count with differential, liver function test, basic metabolic panel, T cell subset analysis, HIV viral load).

HIV-positive individuals were stratified according to CD4 counts. Twenty HIV-positive individuals with CD4>200, referred to as group-1, and twelve with CD4<200 or CD4<14% (HAART naïve), referred to as group-2, received no HAART therapy before immunization. Eleven HIV-positive individuals with CD4 of <200 (HAART treated), referred to as group-3, were treated with HAART for 6–12 months before immunization with PPV23 (includes serotypes 1, 2, 3, 4, 5, 6B, 7F, 8, 9N, 9V, 10A, 11A, 12F, 14, 15B, 17F, 18C, 19F, 19A, 20, 22F, 23F, and 33F). Individuals with CD4<200 were randomly divided into the HAART naïve or HAART treated groups. HAART included two nucleoside analog reverse transcriptase inhibitors and one non-nucleoside reverse transcriptase inhibitor or a boosted protease inhibitor. Twenty two HIV-negative volunteers participated in this study. All volunteers were vaccinated with PPV23 (Merck & CO., INC) on day 0. PPS14 and PPS23F were selected for these studies because of their inclusion in PPV23, differences in capsular polysaccharide configuration and charge, and the ability to correlate with previous studies [23–25]. All adverse events to vaccination were recorded; only 1 patient developed an Arthus-type reaction. All work was conducted in accordance with the Declaration of Helsinki. All volunteers completed the study. We characterized immune responses as indicated by antigen-specific antibody titers, opsonophagocytic titers, and B cell phenotypes.

### Collection of samples

Blood samples were drawn 0 and 30 days post-immunization and subject to ELISA and opsonophagocytic assay. Blood samples drawn 0 and 7 days post-immunization were subject to flow cytometry.

#### Total PPS-specific antibody titers

ELISAs were performed using day 0 and 30 volunteer serum samples along with serum standards 89SF and 007sp. Both control and volunteer serum samples were absorbed with PPS22F and cell wall polysaccharide (CWPS) based on the ELISA training manual published by World Health Organization (WHO) [26]. Briefly, Nunc Maxisorp 96 well plates were coated with 15 μg/ml PPS either 14 or 23F and incubated overnight at 37°C. Absorbed plates were washed with wash buffer (1X PBS, 0.05% Tween 20). After blocking the plates (1X PBS/1% BSA buffer), serially diluted sera were added to the plates and incubated at 37ºC. Plates were washed, and bound antibody was detected using HRP-conjugated anti-human IgG or IgM (Southern Biotech). Plates were developed using o-phenylenediamine substrate and read at 490 nm on a microplate reader. Linear regression fits were used to determine the antibody concentrations and were reported as μg/ml [23]. Representative samples were independently verified by an independent laboratory (ARUP, Salt Lake City, Utah).

#### Functional PPS-specific antibody titers

Opsonophagocytic assay was performed using day 0 and 30 volunteer serum samples as described previously [23,27]. Briefly, serotypes 14 and 23F were incubated with serially diluted heat-inactivated donor sera. Newborn rabbit serum (Pel-Freez, Brown Deer, WI) was added as a source of complement. Differentiated HL-60 cells were added at an E:T ratio of 400:1. The opsonophagocytic titer was defined as the reciprocal of the dilution with 50% killing when compared with serum free controls and analyzed using the Opsotiter1 software developed by the University of Alabama at Birmingham [23,27].

#### Labeling of polysaccharides

Conjugation of PPS14 to cascade blue (CB) ethylenediamine (Invitrogen catalog C-621) or PPS23F to 5-(4,6-dichlorotriazinyl) aminofluorescein (5**-**DTAF; Sigma-Aldrich #36565) was carried out as previously described (Alamo Laboratories Inc, San Antonio, TX) [23].

#### Characterization of PPS-specific B cells

Flow cytometry was performed as previously described [23–25]. Briefly, peripheral blood mononuclear cells were collected from immunized volunteers at 0 and 7 days post-vaccination. Day 7 post-vaccination is when the peripheral blood B cell response peaks allowing analysis [23,28]. Enriched lymphocytes were blocked with PPS22F and CWPS to prevent non-specific labeling with fluorescent PPS. Cells were then labeled with 10 μg/ml of either PPS14-CB or PPS23F-5-DTAF. Fluorochrome-conjugated monoclonal antibodies (BD Bioscience or eBioscience) to the following anti-human Ags were used: CD19 (APC**-**Cy7), CD27 (PerCP**-**Cy5.5), IgM (APC). Cells were analyzed as previously described with FACSAria using FACSDiva software (BD Biosciences) [23,24]. FCS files were analyzed using FlowJo software (Tree Star, Ashland, OR). Populations were divided into three sub**-**populations: naïve (CD27^−^), IgM+ memory (CD27^+^IgM^+^), and switched memory (CD27^+^IgM^−^) B cells. Absolute numbers of B cells were calculated for each donor individually by multiplying the lymphocyte counts by the percentage of B cells determined by flow cytometry.

### Statistical analysis

Pre- and post-vaccination data in a single group were analyzed using paired t-test. Group comparisons were performed using analysis of variance (ANOVA) with Tukey post-hoc test. Pre- to post-vaccination comparisons between groups were calculated by analysis of covariance (ANCOVA) with Bonferroni correction. Correlations between two groups were examined using Pearson correlation. Data is presented as mean ± standard error of the mean (SEM). P-values <0.05 were considered statistically significant. Statistical analysis was performed using Statistical Analysis Software (SAS).

## Results

### Study Population

A total of 65 individuals were enrolled in the study. Their characteristics were described in Table 1 divided by group as described in the methods. Mean age was higher in groups with lower CD4 counts. This is likely reflective of disease progression over time before diagnosis and treatment.

### HIV-positive individuals exhibited diminished PPS-specific antibody titers

**Figure 1:**
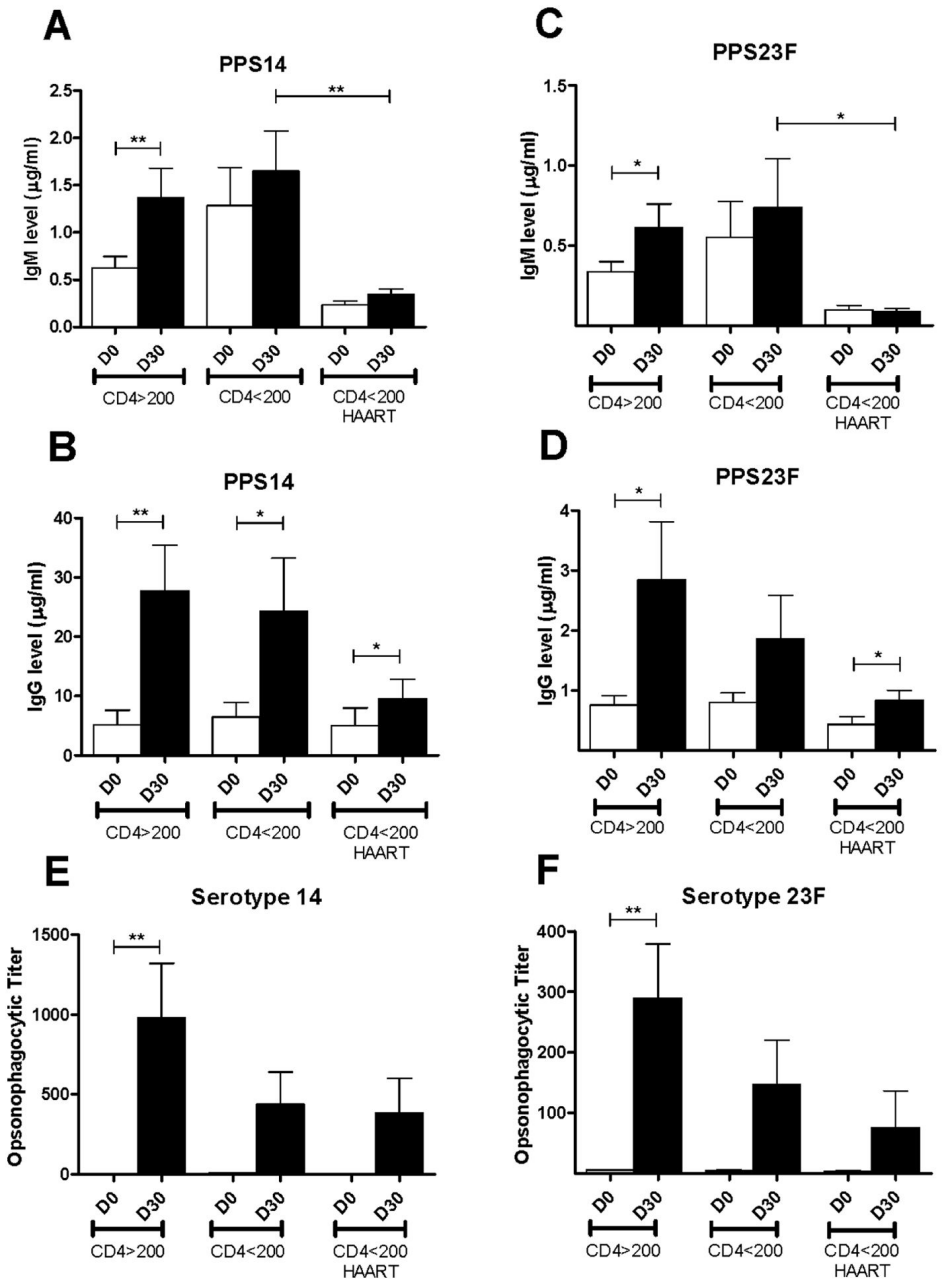
HIV-positive serum antibody and opsonophagocytic titers HIV-positive volunteers were immunized with PPV23. Serum samples were obtained on days 0 and 30 post-immunization. Volunteers were classified according to CD4 count, <200 or >200 cells, and pre-vaccination HAART use. All samples were tested for PPS14 and PPS23F-specific IgG and IgM titers by ELISA expressed as μg/ml (n=43) (A–D) and functional activity by OPA expressed as opsonophagocytic titer (PPS14 n=42, PPS23F n=39) (E–F). Data are mean ± standard error of the mean. *p<0.05, **p<0.01, ***p<0.001.

To evaluate the PPS-specific immune response to PPV23 in newly diagnosed HIV-positive individuals, day 0 and day 30 post-vaccination sera were collected. Samples were tested by ELISA to determine PPS-specific antibody concentrations. Pre- to post-immunization, group-1 (CD4>200) showed a significant increase in PPS14-specific IgM from 0.6 ± 0.1 μg/ml to 1.4 ± 0.3 μg/ml (p<0.01) and IgG from 5.2 ± 2.5 μg/ml to 27.8 ± 7.7 μg/ml (p<0.01) (Figure 1A, 1B). Volunteers in group-2 (CD4<200, HAART naïve) showed no significant increase in PPS14-specific IgM from 1.4 ± 0.4 μg/ml to 1.8 ± 0.4 μg/ml, however, IgG increased significantly from 6.9 ± 2.7 μg/ml to 26.0 ± 9.7 μg/ml (p<0.05). Similarly, group-3 (CD4<200, HAART treated) showed no significant increase in PPS14-specific IgM from 0.2 ± 0.0 μg/ml to 0.3 ±0.1 μg/ml, however IgG increased significantly from 5.0 ± 3.0 μg/ml to 9.6 ± 3.3 μg/ml (p<0.05).

Group-1 PPS23F-specific IgM increased significantly from 0.3 ± 0.1 μg/ml to 0.6 ± 0.1 μg/ml (p<0.05) and IgG from 0.8 ± 0.2 μg/ml to 2.9 ± 1.0 μg/ml (p<0.05) (Figure 1C, 1D). Group-2 showed no increase in PPS23F-specific IgM from 0.6 ± 0.2 μg/ml to 0.8 ± 0.3 μg/ml or IgG from 0.8 ± 0.2 μg/ml to 1.9 ± 0.8 μg/ml. No increase occurred in group-3 PPS23F-specific IgM which remained at 0.1 ± 0.0 μg/ml, but IgG increased significantly from 0.4 ± 0.1 μg/ml to 0.8 ± 0.2 μg/ml (p<0.05). PPS14 and PPS23F IgM and IgG levels post-immunization were lower in group-3 (HAART treated) compared to group-2 (HAART naïve), however, only PPS14- and PPS23F-specific IgM were statistically significant (p<0.01 and p<0.05 respectively). No significant differences between HIV-positive groups in their response to vaccination were found. Overall, HIV-positive individuals demonstrated low PPS-specific antibody concentrations post-vaccination.

### HIV-positive individuals exhibited diminished PPS-specific functional antibody responses

PPS-specific opsonophagocytic assays were performed following the standardized protocol developed by Nahm et al. to test the functional ability of antibody to enhance phagocytic clearance of serotype specific pneumococcus [26]. Group-1 demonstrated a significant increase in OPT post-immunization against serotype 14 (3.4 ± 0.4 to 980 ± 340, p<0.01) and serotype 23F (5.3 ± 1.1 to 290 ± 90, p<0.01) (Figure 1E,1F). Group-2 showed non-significant increases in OPT against serotype 14 (9.5 ± 2.8 to 440 ± 201) and serotype 23F (4.5 ± 1.8 to 147 ± 74). Similarly, group-3 showed non-significant increases in OPT against serotype 14 (3.4 ± 0.7 to 385 ± 215) and serotype 23F (3.9 ± 1.1 to 75 ± 61). Each donor, however, demonstrated a minimal 2-fold increase in OPT post-vaccination and all donors had an OPT >8, thought to correlate with immune protection, despite low IgM and IgG antibody concentrations [29]. No significant differences between HIV-positive groups in their response to vaccination were found. Overall, all groups showed post-vaccination OPT well above 8 despite non-significant increases in antibody titers from pre- to post-vaccination.

### Decreased B lymphocyte populations in HIV-positive peripheral blood

Unselected CD19+ B cell percentages and numbers in the peripheral blood of HIV-positive individuals were analyzed and compared to that of HIV-negative individuals (Table 1). The total B cell percentages and absolute number of all groups were not significantly different as determined by ANOVA.

The phenotypes of unselected B cells were similar on day 0 and day 7 in all groups (data not shown). Analysis of unselected B cells primarily showed no significant differences in distribution of B cell phenotype between the HIV-negative, HIV-positive with CD4>200 and HAART naïve. Specific statistical differences are noted in Table 1. Moreover, the HAART treated group had a significantly lower percentage and number of IgM+ memory and switched memory B cells compared to HIV-negative individuals as determined by ANOVA. B cell percentages and numbers were not significantly different between the HAART naïve and HAART treated groups.

### Phenotypic analysis of PPS-specific B cells in HIV-positive peripheral blood

To determine the effect of immunization with PPV23 on the B cell populations circulating in the peripheral blood, PPS-selected B cells were analyzed by flow cytometry as previously described [23–25]. An example of the gating used for phenotypic analysis is shown in Supplemental Figure 1. The percentage and number of PPS-selected B cells increased significantly from day 0 to day 7 for all groups and both PPS (p<0.01) (Table 2). No significant differences in percentage or number of PPS-specific B cells between HIV-positive groups were found for either PPS.

**Figure 2:**
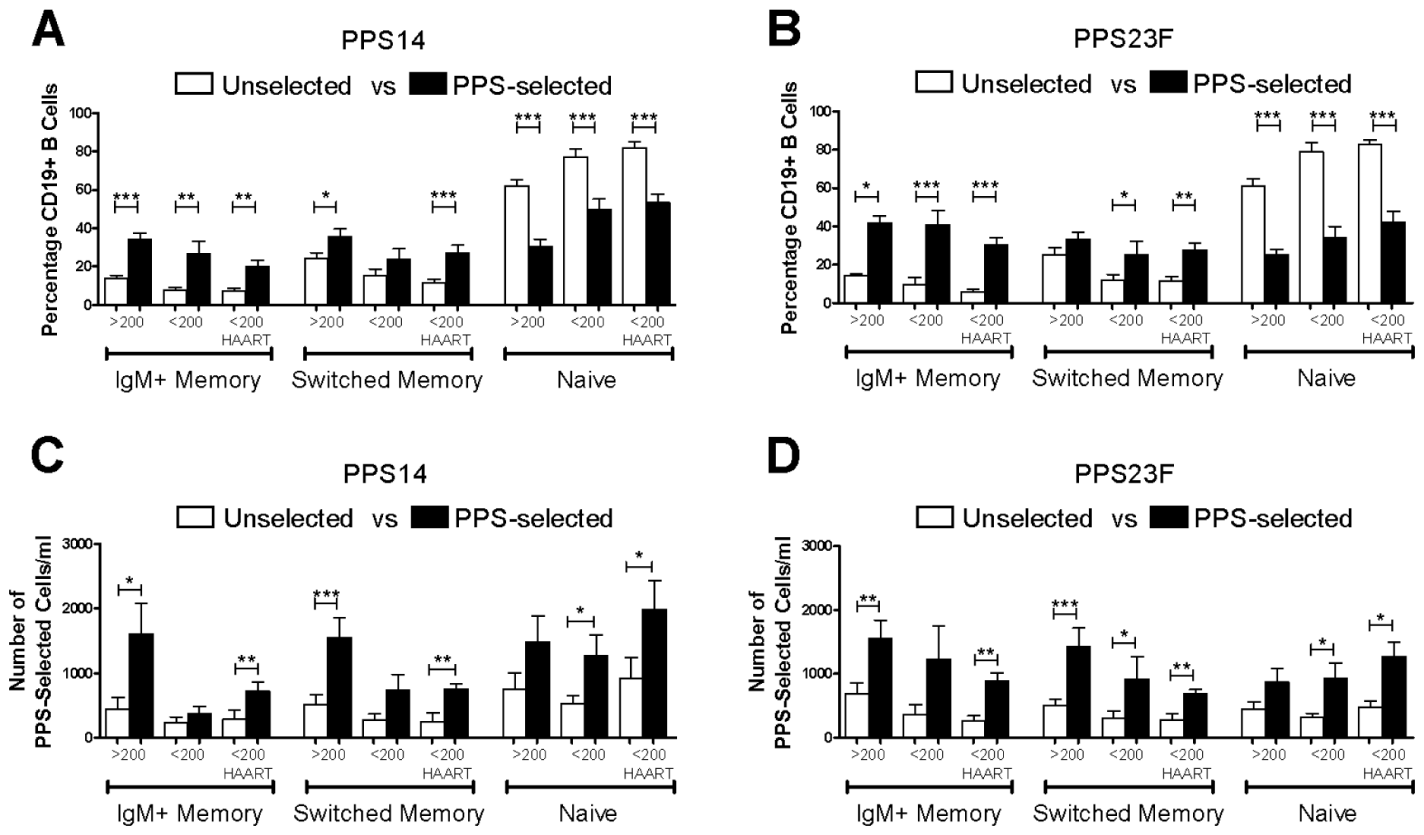
PPS-specific B cells from HIV-positive individuals immunized with PPV23 HIV-positive donors were immunized with PPV23. On day 0 and day 7 post-immunization, lymphocyte enriched peripheral blood samples stained with fluorescently labeled PPS14 or PPS23F were evaluated by flow cytometry for distribution of CD27 and IgM among PPS-selected CD19+ B cells. Results for all HIV-positive individuals were categorized by CD4 counts and HAART usage. B cells were expressed as a percentage of total unselected or PPS-selected CD19+ B cells (A,B). B cells were expressed as the number of PPS-selected CD19+ B cells per ml (C,D). In each sample, 100,000 events were recorded. Data are mean ± standard error of the mean. *p<0.05, **p<0.01, ***p<0.001.

The majority of unselected B cells were naïve cells (Table 1). Overall, the phenotype distribution of PPS-selected cells on day 7 was significantly different from that of unselected cells (Figure 2A,2B). All HIV-positive groups showed significant increases in the percentage of PPS14-selected IgM+ memory B cells compared to unselected cells [group-1 14.0 ± 1.3 to 34.3 ± 3.2, group-2 7.7 ± 1.5 to 26.4 ± 6.5, group-3 7.0 ± 1.6 to 20.5 ± 3.4] (Figure 2A). Group-1 and group-3 showed significant increases in the percentage of PPS14-selected switched memory B cells compared to unselected cells [group-1 24.1 ± 3.1 to 35.4 ± 4.2, group-2 15.1 ± 3.6 to 23.9 ± 5.4, group-3 11.3 ± 2.1 to 26.8 ± 4.3]. PPS23F selection generally showed similar differences compared to unselected cells [IgM+ memory; group-1 14.1 ± 1.3 to 41.8 ± 3.7, group-2 9.5 ± 3.7 to 40.8 ± 7.3, group-3 5.9 ± 1.1 to 30.2 ± 3.8] [switched memory; group-1 25.2 ± 3.7 to 33.3 ± 3.7, group-2 11.7 ± 3.3 to 25.1 ± 6.9, group-3 11.6 ± 2.2 to 27.6 ± 3.6] (Figure 2B). There were no significant differences between HIV-positive groups regarding the percentage of PPS-specific subgroups.

Groups 1 and 3 showed significant increases from day 0 to day 7 in the absolute number of PPS14-selected IgM+ memory B cells (group-1 449 ± 175 to 1605 ± 470, group-2 237 ± 83 to 366 ± 121, group-3 286 ± 137 to 720 ± 146) (Figure 2C). Similar increases were seen for the number of PPS14-selected switched memory compared to unselected B cells (group-1 514 ± 147 to 1548 ± 316, group-2 275 ± 96 to 736 ± 246, group-3 250 ± 129 to 751 ± 90). PPS23F selection showed significant increases in PPS-selected IgM+ memory B cell numbers from day 0 to day 7 for groups 1 and 3 (group-1 688 ± 169 to 1555 ± 282, group-2 366 ± 147 to 1225 ± 524, group-3 259 ± 85 to 880 ± 132). PPS23F-selected switched memory B cells increased significantly for all groups (group-1 509 ± 99 to 1430 ± 283, group-2 (303 ± 112 to 920 ± 348), group-3 (278 ± 93 to 688 ± 66) (Figure 2D). These data show that IgM+ memory and switched memory B cells significantly increase in response to PPV23 in newly diagnosed HIV-positive individuals. No significant differences between HIV-positive groups regarding the number of PPS-specific subgroups were found.

### Diminished immune response to PPV23 in HIV-positive compared to HIV-negative individuals

HIV-negative individuals were directly compared to HIV-positive groups to identify deficiencies in serological responses. HIV-negative individuals showed significant increases in IgM and IgG levels and OPT both pre- and post-vaccination (Supplemental Figure 2) [23,24]. HIV-negative individuals post-vaccination showed significantly higher PPS14-specific IgM (12.2 ± 0.2 μg/ml), PPS23F-specific IgM (8.9 ± 0.1 μg/ml), and PPS23F-specific IgG (13.8 ± 0.3 μg/ml) titers compared to HIV-positive groups. Conversely, post-vaccination PPS14-IgG levels (35.2 ± 0.4 μg/ml) were not significantly different between HIV-negative and HIV-positive groups. OPT of HIV-negative individuals were significantly higher post-vaccination compared to HIV-positive groups for both PPS14 and PPS23F serotypes. All HIV-positive groups had significantly lower responses to vaccination compared to HIV-negative individuals.

**Figure 3:**
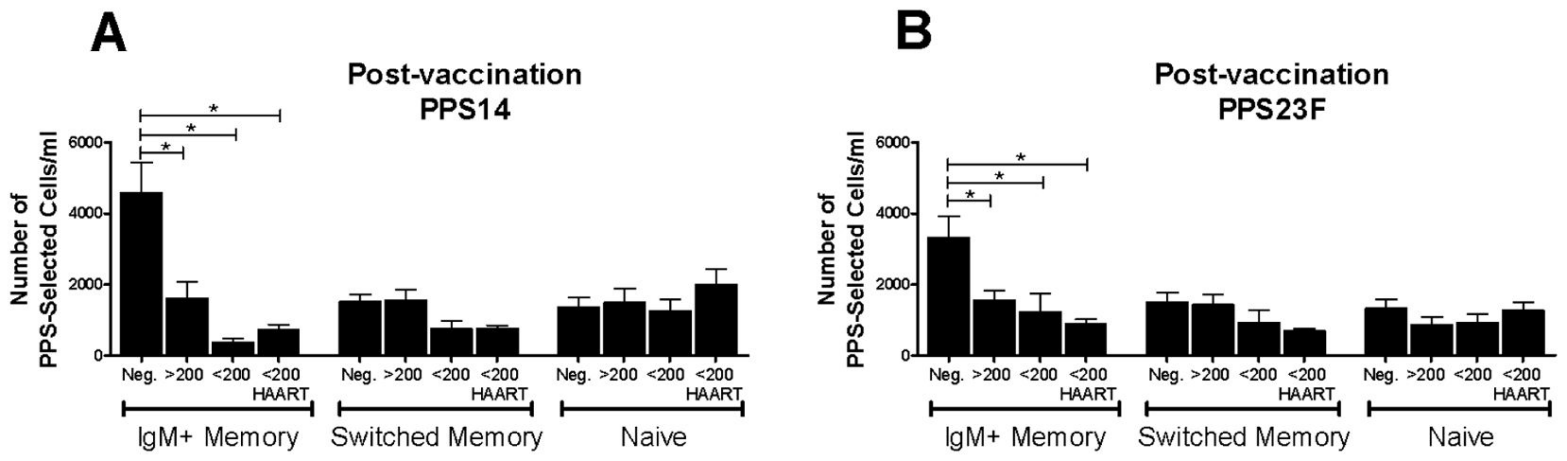
HIV-positive donors show significantly diminished response to PPV23 compared to HIV-negative donors HIV-positive donor samples were compared to HIV-negative donor samples day 7 post-immunization with PPV23. Lymphocyte enriched peripheral blood samples stained with fluorescently labeled PPS14 or PPS23F were evaluated by flow cytometry for distribution of CD27 and IgM among PPS-selected CD19+ B cells. Post-immunization results for all HIV-positive individuals were categorized by CD4 counts and HAART usage. B cells were expressed as the number of PPS-selected CD19+ B cells per ml (A,B). In each sample, 100,000 events were recorded. Data are mean ± standard error of the mean. *p<0.05.

HIV-negative individuals were similarly compared to HIV-positive groups to identify deficiencies in PPS-selected B cell numbers. Post-immunization, HIV-negative individuals had significantly higher numbers of PPS-selected IgM+ memory B cells (PPS14: 4561 ± 805 cells/ml, PPS23F: 3308 ± 611 cells/ml) compared to each HIV-positive group (HIV-positive data reported in Figure 2C, 2D) (p<0.05) (Figure 3A, 3B). No significant differences were seen between IgM+ memory cells in HIV-positive groups. No significant differences between the number of PPS14- or PPS23F-selected switched memory cells was noted between HIV-negative (PPS14: 1524 ± 208 cells/ml, PPS23F: 1495 ± 284 cells/ml) and HIV-positive individuals. HIV-positive individuals have significant deficiencies in their PPS-specific IgM+ memory B cell response compared to HIV-negative individuals including those with CD4>200. Six to twelve months of HAART had no significant effect on the number of PPS-selected cells in individuals with CD4<200.

## Discussion

We compared the response to PPV23 in HIV-positive individuals, stratified according to CD4 count as a surrogate marker for disease progression. In agreement with previous studies, our HIV-positive population showed diminished polysaccharide-specific IgM and IgG titers post-vaccination compared to reports of HIV-negative individuals [8,9,22–24]. It has been postulated that control of HIV viral load, leading to reduced B cell hyperactivation, may improve B cell function and result in increased PPS-specific antibodies. Our HIV-positive individuals with CD4<200 receiving HAART for 6–12 months pre-immunization, paradoxically demonstrated lower PPS-specific antibody levels post-vaccination compared to HIV-positive individuals with CD4<200 without HAART. To confirm this observation, antibody titers of representative samples were independently verified and corresponded well with our results and with previous reports both pre- and post-PPV23 [9,30,31]. Our PPS-specific antibody concentrations of HIV-positive individuals correlated poorly with CD4 counts and OPT, suggesting that PPS-specific antibody concentrations serve as a poor clinical surrogate marker for immune protection [21,29]. High levels of antibodies in individuals with CD4<200 may result from chronic viral infection induced inflammation and high viral titers non-specifically stimulating PPS-binding, but non-functional, antibodies. Removal of viral non-specific stimulation with HAART usage likely reduces production of non-functional antibodies [32]. Furthermore, HIV infection may result in the progressive loss of PPS-specific B cells which may become permanently lost and/or functionally inhibited and cannot restore antibodies levels after 6–12 months of HAART [13,33]. This may explain why increases in PPS-specific B cell numbers do not correspond with antibody levels or OPT in individuals with CD4<200 as a result of long term infection with chronic inflammation [29,34,35].

Functional impairment of antibodies in HIV-positive individuals has also been reported [35]. Opsonophagocytic assays are a more sensitive and reliable indicator of vaccine response than polysaccharide based ELISA. Analysis of OPT is not confounded by the presence of non-functional antibodies produced from HIV infection. HIV-positive individuals with CD4>200 showed a significant increase in OPT post-immunization, but increases in individuals with CD4<200 were non-significant. This highlights the importance of early control of HIV infections. Regardless of CD4 counts, OPT levels were diminished compared to previous reports of HIV-negative individuals, demonstrating humoral immune deficiency beginning at an earlier stage of infection [23,24]. HAART treatment for 6–12 months did not improve functional opsonophagocytic titers suggesting that there was no benefit in delaying vaccination.

Equivalent levels of antibody titers between individuals with CD4>200 and CD4<200 for PPS14 did not correlate with equivalent OPT. Additionally, IgG titers were lower and IgM titers decreased significantly in individuals receiving 6–12 months HAART. However, this was not true for OPT, suggesting that virological control in individuals with CD4<200 resulted in a reduction of non-functional antibodies. This is supported by previous reports which also suggested that low correlations between antibody titers and OPT among the HIV-positive population may be due dysfunctional antibodies in some individuals [29,35]. Low antibody titers and OPT likely result from considerable B cell dysfunction [11,36]. Regardless, an OPT>8 is thought to correlate with immune protection suggesting that PPV23 vaccination was beneficial for all donors regardless of CD4 count [29]. These data will serve as an important basis for comparison to evaluate new vaccination methods including the recent recommendations to include the 13-valent pneumococcal conjugate vaccine.

HIV-positive individuals showed a similar percentage and number of total B cells compared to HIV-negative individuals. The slight increase in the percentage of B cells in HIV-positive individuals with CD4<200, compared to those with CD4>200, in contrast to the absolute number of B cells/μl, likely reflects the loss of T cells shifting the proportion of total lymphocytes. HAART treatment for 6–12 months had no effect on total B cell percentages or numbers suggesting that cellular restoration in individuals with CD4<200 requires considerable time after viral suppression [12,37,38].

The cells responsible for producing anti-PPS specific antibodies is a controversial issue [39–43]. Several previous studies have analyzed unselected cell populations post-immunization, however it is unlikely that these studies accurately represent the small antigen-specific population (<1–4%) [13,38]. We have demonstrated the ability to identify PPS-specific B cells using fluorescently-labeled polysaccharide in conjunction with flow cytometry. The specificity of our labeled PPS was supported by inhibition assays and the ability to bind to PPS-specific monoclonal cells [23]. Likewise, binding of labeled PPS to post-vaccination peripheral blood B cells in flow cytometry was inhibited with addition of homologous unlabeled polysaccharide. We have previously shown in HIV-negative individuals, who respond well to PPV23, that the IgM+ memory population significantly increased post-vaccination and constituted the majority of PPS-specific B cells responding to vaccination 7 days post vaccination and correlates well with OPT levels [23,24].

In this study, we directly characterized the PPS-specific B cells responding to PPV23 in newly-diagnosed HIV-positive individuals and compared them to those from HIV-negative individuals. Our analysis of B cell numbers showed that both PPS-selected IgM+ memory and switched memory B cells increased significantly post-immunization, but generally tended to show lower levels with low CD4 counts. Post-immunization levels of PPS-selected switched memory B cells in HIV-positive individuals were comparable to those in HIV-negative individuals. However, IgM+ memory cells were significantly reduced in HIV-positive individuals. Overall, HIV-positive individuals only demonstrated significantly reduced PPS-specific IgM+ memory B cell numbers compared to HIV-negative individuals, thus identifying a specific cellular deficiency in the immune repertoire and may be responsible for humoral deficiencies against *Streptococcus pneumoniae*. The correlation between loss of IgM+ memory B cells and decreased humoral response is in agreement with previous publications which showed that individuals with decreased IgM+ memory B cells such as splenectomized, infants <2 years old, and the elderly respond poorly to PPV23 [40,44,45]. Moreover, individuals treated with HAART for 6–12 months pre-vaccination showed no significant increase in the number of PPS-selected IgM+ memory or switched memory B cells post-immunization. This is possibly related to the delay in B cell reconstitution following the institution of anti-retroviral therapy [12,37,38].

These antigen-specific antibody titers, OPT, and B cell studies emphasize the importance of early clinical HIV infection diagnosis, virologic control and immediate immunization. While it remains beneficial to immunize individuals with CD4<200 based on OPT results post-immunization, immune responses deteriorate in patients with lower CD4 counts. Our study suggests that despite significantly lower viral titers after 6–12 months treatment with HAART, it did not result in improved OPT, most likely the best correlate of protection. Based on these findings, vaccination should not be delayed in newly diagnosed HIV-positive individuals with CD4<200.

To our knowledge, we are the first to examine antigen-specific B cell subpopulations and serological effects of 6–12 months of HAART before vaccination with PPV23 in newly diagnosed HIV-infected individuals with CD4<200. A larger sample size may increase the power of analysis sufficiently to distinguish between HIV-positive groups, but would require enrollment of thousands of individuals. Additional serotypes may show alternative responses in HIV-negative and HIV-positive individuals but there are practical limitations to these studies. Future studies will focus on the response of newly diagnosed HIV-positive individuals to alternative vaccination strategies including the recently approved 13-valent pneumococcal conjugate vaccine.

## Supplementary Material

Supplementary file

## Figures and Tables

**Table 1:** Study population and unselected CD19+ B cell percentages and counts.

Parameter	HIV-negative	CD4>200	CD4<200	CD4<200, HAART
n-value	22	20	12	11
Age (mean/range)	26/21–33	28/19–46	36/20–57	45/35–59
Gender (M/F)	13/9	19/1	11/1	11/0
Ethnicity(A/B/H/W)	10/0/0/12	0/13/0/7	0/7/0/5	0/3/2/6
HAART	-	-	-	6–12 months
Pre-HAART viral load	-	-	-	505,476 ± 230,034
Day 0 viral load	0#	47,160 ± 25,845#	1,066,177 ± 515,031	12,158 ± 12,011#
Nadir CD4	-	553 ± 54*,#	109 ± 26	69 ± 23
Vax CD4, #/μl [%]	-	553*,# [30.5]*,#	126 [7.8]	206 [12.6]
CD19+ B cells, %	9.5 ± 0.6	8.7 ± 0.8	12.4 ± 1.9	9.2 ± 1.3
CD19+ B cells, #/μl	166.5 ± 21.5	166.5 ± 19.3	127.3 ± 13.3	114.0 ± 19.3
**Unselected CD19+ B cell subsets, %**				
IgM+ Memory	12.5 ± 1.1*	13.5 ± 1.6*	7.9 ± 2.7	5.8 ± 1.2
Switched Memory	16.3 ± 1.6*	15.9 ± 2.0*	10.0 ± 3.2	7.0 ± 0.9
Naïve	71.2 ± 2.5*	70.6 ± 2.7*	82.0 ± 4.6	87.2 ± 1.5
**Unselected CD19+ B cell subsets, #/**μ**l**				
IgM+ Memory	17.2 ± 2.1*,#	22.0 ± 3.3*	11.1 ± 3.2	8.4 ± 2.6
Switched Memory	24.4 ± 6.5*	30.3 ± 6.9	12.5 ± 4.6	7.4 ± 1.3
Naïve	124.9 ± 16.5	114.2 ± 12.6	103.7 ± 14.1	98.2 ± 13.7

Viral loads are reported as copies/ml. Vax: Value at time of vaccination, A: Asian, B: Black, H: Hispanic, W: White. Data are mean ± standard error of the mean.

* indicates significant difference (p<0.05) compared to the CD4<200, HAART group.

# indicates significant differences (p<0.05) compared to the CD4<200 group.

**Table 2:** Pneumococcal polysaccharide-selected CD19+ B cell percentages and counts among HIV-positive individuals before and 7 days after PPV23 immunization. CD19+ PPS+ B cells.

**Percentage of CD19+**	PPS14+ D0	PPS14+ D7	PPS23F+ D0	PPS23F+ D7
CD4>200	0.9 ± 0.2	2.7 ± 0.4	0.9 ± 0.2	2.5 ± 0.3
CD4<200	0.8 ± 0.2	2.2 ± 0.5	0.8 ± 0.2	2.3 ± 0.5
CD4<200 + HAART	1.2 ± 0.4	3.5 ± 0.7	0.9 ± 0.2	3.2 ± 0.6
**Number per ml**	PPS14+ D0	PPS14+ D7	PPS23F+ D0	PPS23F+ D7
CD4>200	1716 ± 470	4634 ± 981	1652 ± 293	3854 ± 622
CD4<200	1046 ± 270	2370 ± 545	990 ± 280	3066 ± 855
CD4<200 + HAART	1448 ± 570	3456 ± 526	1015 ± 260	2834 ± 252

Data are mean ± standard error of the mean.
